# Plain Talk about Early Diagnosis and Early Operation in Certain Intrapelvic Diseases of Women1Read by invitation before the Medical Society of the County of Erie, at Buffalo, June 14, 1892.

**Published:** 1892-09

**Authors:** Charles A. L. Reed

**Affiliations:** Cincinnati, O.; Fellow of the American Association of Obstetricians and Gynecologists; 311 Elm Street


### PLAIN TALK ABOUT EARLY DIAGNOSIS AND EARLY OPERATION IN CERTAIN INTRAPELVIC DISEASES OF WOMEN.[1]

By CHARLES A. L. REED, M. D., of Cincinnati, O.

Fellow of the American Association of Obstetricians and Gynecologists.

The chief cause of mortality in abdominal and pelvic surgery may be epitomized in the word "delay." Papers by eminent specialists emphasizing this point, are to be found in almost every journal, while case reports giving pictures but little less than tragic, stand as danger signals upon almost every page of gynecic literature. The question of responsibility, in such instances, is a most serious one. Who is responsible for the delay? The patients themselves are frequently alone to blame. There is a natural timidity, an inborn modesty, about woman which comprises her loveliest charm, but which at once becomes converted into immodesty the moment it is permitted to establish a barrier to her well-being. This immodesty is displayed whenever a woman declines proper investigation of her case in the presence of persistent symptoms pointing to disease within the pelvis. She should remember that it is the business of physicians to investigate such cases, and in the discharge of their duty in this regard, it should be remembered also that they, too, have feelings to sacrifice.

There are cases in which the delay in submitting to an operation is to be attributed to the cowardice of patients who may have submitted to examination, and who may have been properly advised by their attending physicians. It is but natural that any woman should shrink from a surgical operation, at least until she can intelligently appreciate the alternative. If, however, we seek to trace this apprehension of results, we shall find the source to be in a multitude of conditions, some, if not all, of which point to evils within the profession, but evils which, so far as the individual practitioners are concerned, are easily avoidable. There is hardly a woman afflicted with an intrapelvic condition demanding an operation but who has heard of some death following such an operation, and is deterred from availing herself of the resource of surgery purely out of apprehension of a similar result. It is sufficient for her that a woman was ill, was operated upon, and died. She does not go back of this trio of facts. If she were to go back of them, she would find, very probably, that the disastrous result had occurred

1. Read by invitation before the Medical Society of the County of Erie, at Buffalo, June 14, 1892.

from an amateurish exploit in surgery at the hands of some one who had never served an apprenticeship at his calling, or else, after long delay, she had fallen victim to complications which had developed through her long-continued and worse than futile procrastination,—complications which baffle the skill of the most sagacious of surgeons and set at defiance every resource of our art.

Many women are deterred from timely operation by a misapprehension on the part of their medical attendants of the true status of pelvic surgery. It was but recently that a patient came to me with a pelvis full of pus, and when I told her that she must submit at once to an abdominal section for her relief, she informed me that she had no hope of recovery, as her physician had told her that three-fourths of all women operated upon in that way died, and that, in consequence of that statement, she had been deferring operation for nearly three years, during all of which time she had been growing worse. This is one extreme illustration of the ignorance sometimes displayed by members of a profession which is, to my mind, the best informed and most conscientious of all the professions.

What is needed to remedy this evil is to have the actual results of special workers in this field of surgery published and republished, until the general profession begins to comprehend what can be done for the relief of their unfortunate patients. It should be held up that the mortality of abdominal section, in skilled hands, is under three per cent., and that, with all operators, results improve just in proportion as they get their cases early and as their experience increases.

It has finally grown to be a conviction with the profession that only one thing ought to be done with an ovarian tumor, and this conviction is shared by the laity,—indeed all deep professional convictions are at once reflected upon the people. The time was not long since when it was the advice of physicians to their patients having ovarian tumors, "Do not bother the tumor until the tumor bothers you." The primary mortality from ovariotomy, during that period, was, naturally enough, from twenty-five to forty per cent. The profession, with its natural conservatism, was slow to grasp the truth taught by these tragic figures. We are now living, however, in a happier epoch. "Operate for ovarian tumors as soon as they can be detected," is the mandate of surgery which is being echoed by every physician in the land who does his duty by his patients. What is the result? The mortality from ovariotomy has become practically *nil.* In my own work I did not lose a single case from ovariotomy in the Woman's Surgical Hospital of Cincinnati since its opening, two and a half years ago, until the very last one,—a sadly neglected case of three years' standing, from which I removed over sixty pounds of tumor, solid and fluid, and who died of exhaustion due to a frightful serous flow from a chronically inflamed peritoneum. What is true of my own work in this particular, is true of that of other operators of wider experience. The profession has taken a distinct stand in the question of the removal of ovarian tumors,—a stand which is saving more life than any doctrine aside from that of asepsis, in the whole range of surgery. What the profession has done with regard to ovarian cystomata should be done with regard to all strictly surgical conditions within the pelvis. Early operation should become the shibboleth of the conscientious practitioner.

And now I desire, just here, to interpolate a paragraph for the sole purpose of saving me and those who speak as I have just spoken from misinterpretation and misrepresentation. I ask you to note that I insisted upon examination only in the "presence of persistent symptoms pointing to disease within the pelvis." The word "persistent" used in this connection is a significant one, and implies that conservative measures of treatment have first been tried, unless the subjective phenomena have been such as to place all temporizing beyond reasonable practicability. In the case of virgins, examination should be practised only in the presence of imperative indications. I have been referred to in the journals as one who operates upon everything right away,—and my colleagues (Drs. Price, McMurtry, and Ross,) who are addressing you upon this occasion have been subjected to similar criticisms. Allow me to say that I do not operate upon twenty-five per cent. of the cases that come to me for the especial purposes of operation, and that I never decline to operate upon any case that in my judgment can be best remedied by operation, or that has a remaining chance of recovering from surgical interference. I am sure that my colleagues are equally discriminating in accepting their cases, but when we do accept cases for operation we very much desire that they shall yet be within the operable list. If we are to get these cases when they are yet remediable, if we are to get them when their severer complications have not yet developed, we must look to the general practitioner for coöperation, who is first called for their relief. And I may go a step further and say that we will get them from him at this desirable stage and in this desirable condition only when due consideration is given to early diagnostic indications.

General practitioners, particularly in the smaller towns and rural districts, labor under certain disadvantages in assuming charge of these cases. Twelve years' experience in rural practice enables me to speak knowingly upon this point. I have already alluded to what is to my mind the chief of these disadvantages, namely, the indisposition of patients to submit to examination. If, however, the patient be not a very ignorant woman, she will generally acquiesce when plainly, intelligently, and firmly but gently told of the serious possibilities arising out of delay, and the utter futility, not to say hazard, of any sort of treatment without a preliminary diagnosis. With the patient's consent to make an examination, care ought to be taken that certain things should not be done. A writer in Mann's System of American Gynecology advises that at every first interview the external genitalia should be subjected to careful inspection, and offers certain alleged reasons why this should be done. There is no more reason why this should be done than why a woman should be ushered into the consultation room with the bust exposed. The procedure is as indelicate as it is unnecessary, and ought never to be practised in the absence of some special indication. No instrument should be used that is not necessary for the detection or treatment of diseased conditions, and should then be selected with reference to ease and painlessness of application. Under this rule, and with our refined armamentarium to choose from, I would no sooner think of making an examination with, for instance, a Fergusson's cylindrical speculum, than with a lamp's flue or a joint of stove pipe. It is this instrument with its tortures that accounts for so many cases never passing beyond their first examination in a course of seriously needed treatment. The sound is today the opprobrium of the gynecic outfit, capable of doing a vast amount of mischief and furnishing no information that cannot be more easily and more accurately acquired through other means, hence, ought not to be employed. I am not now the possessor of such an instrument. It is not necessary for purposes of diagnosis to stand a patient on her head, nor to put her in any of the extraordinary positions so graphically pictured in the books. I cannot imagine anything more repugnant to a refined woman than to come into a strange doctor's office and be made to assume a series of grotesque and utterly needless postures as if she were a professional all-around athlete.

With these "don'ts" well in mind, let us inquire as to the significance of certain facts, subjective and objective, which should prompt us to a diagnosis. The history of the case is important. Permit me to dismiss this branch of my subject by copying from my note book an entry, which I made only yesterday, and which, to my mind, illustrates in a very forcible way what may always be taken as "significant" facts.

Mrs. G., *æt.* 33, menstruated first at 15, sustained a fall; menstruation ceased for fifteen months, when it recurred with extreme pain. She married at twenty; two years later, after two months' cessation of menstruation, she had "a flooding spell," from which she convalesced slowly. She went to the Adirondacks, and rode eighteen miles in a jolt wagon; stopped on her way out of the mountains and had a Fowler pessary inserted. This had to be removed after she got home, because of intense pain. A few years later, not again conceiving as was her desire, she was treated to a series of sponge tents, which caused intense and diffuse pelvic pains; after four months she had some more sponge tents followed by peritonitis, from which she got out of bed only after nine weeks. Some time later she consulted a gynecologist of national reputation, who inserted some other sort of a pessary which she wore for nearly a year, when it was removed by a very intelligent practitioner, who described to me a deep erosion which it had produced on the posterior wall of the cervix. She then got into the hands of still another practitioner, who sought to relieve her by intrapelvic massage *a la* Brandt. This failing, she passed under the care of yet another practitioner, who employed both galvanism and faradism for her cure. This brought her to my consultation room, worn and haggard by her unsatisfactory and exasperating experience, and by the constant dragging within the pelvis. On careful examination, the only condition that I could clearly make out was a retroversion with adhesion. She complained of pain to the touch upon either side of the uterus, particularly upon the right, but I could find neither ovary.

She enters my hospital tomorrow for a little preliminary treatment, when I shall operate upon her. What shall I find? I have done too much pelvic surgery to venture a prediction. There are some things aside from the displaced uterus that cannot help being there. With the history which she has given she cannot avoid adherent ovaries with more than probable occlusion of the tubes,—a condition which destroys her fecundity and imposes upon her the pain which is rapidly reducing her to the state of confirmed invalidism.

In cases whose history has presented " significant facts " such as have been outlined in the preceding record, what are we to look upon as the indications for early operation ? A full discussion of this question would imply the delineation of the early history of every surgical condition occurring within the pelvis. Such a dissertation is not called for in the presence of such an audience as that which now confronts me. Chronic adhesive oöphoritis, acute interstitial salpingitis, acute occlusive salpingitis, chronic hydrops, and follicular ovaritis, are all conditions that cannot be cured short of surgical interference, and yet are conditions that may yield the most negative results on physical exploration even under anesthesia. Their most important symptom is pain,—pain within the pelvis,—generally referred to one side or the other, or to both iliac regions. This pain may find expression in the sacral region also, and later in reflexes down the thighs, up the spine, or in distress suggestive of engorgement of the longitudinal sinus, or in an aggregation of all of these conditions. Pain occurring as a result of any of these pathological states is of the most persistent type, and by constantly nagging the nervous system of the patient, interferes with her physical functions. Digestion is impaired, the bowels become constipated, tympany is persistently present, the bladder is irritable, and the rectum is a constant source of annoyance. With the supply of nutrition cut off through the stomach, with the assimilative functions impaired through intestinal atony, with sleep disturbed if not actually dissipated by constant pain, the trophic centers presently yield, and the patient enters upon a rapid decline. Relief is now sought by anodynes, and in the majority of cases the opium habit is established. With yearnings for maternity which cannot be gratified, with longing for connubial relations which are prohibited by pain, with pathetic prayers for a peace and comfort which are unattainable, these cases go from bad to worse, until the body becomes reduced and, in some instances, the mental and moral faculties become disturbed, if not destroyed.

Let me further illustrate :

A patient, *æt.* 40, came to me a few weeks since. She had had one child fifteen years ago. She had not since been pregnant. During that time she had had pain of a persistent sort in both iliac regions. She was very anxious for children. This desire probably had something to do with the development of a mild nymphomania. She was an excellent woman, and this impulse had kept her in a constant hand-to-hand conflict with herself. She had never transgressed, and came to my hospital with an earnest desire to escape from the torments of a desire which had been in constant quarrel with her higher sense. She had been in sanitoria in the East and in the West without relief. I examined her most carefully under anesthesia with entirely negative results. The persistence of pain, however, taught me that she had a center of irritation within the pelvis, which center of irritation was, to my mind, the cause of the constant sexual perturbation. With this theory in mind, I advised exploratory incision,—a suggestion which was eagerly accepted. In the presence of her attending physician, Professor D. S. Young, of Cincinnati, I opened the abdomen with the result of finding a double hydrosalpinx, each tube being adherent so high up in the pelvis as to elude the touch from a bimanual examination practised under anesthesia. The patient made a prompt recovery, with the additional fact that she is now in a state of mental tranquility. She no longer yearns for a maternity which she now knows is beyond the possibility of attainment.

I mention this case because it is an additional instance of the happy outcome of an operation undertaken on symptoms alone, without the indications of a physical character such as are in some instances solely relied upon as the criteria for surgical interference. I have gratification in presenting it upon this occasion, because it is but another example of the relief of mental conditions by the removal of peripheral irritation,—a principle of treatment for which I had the honor to contend in this hall more than three years ago, and an example which is receiving ample confirmation at the hands of my distinguished friend, Dr. Geo. H. Rohé, at the Maryland hospital for the insane.

It would be futile for me to attempt to discuss all the conditions which should prompt the medical attendant to advise early operation. Their conditions are amplified in detail in the modern text-books, notably in the recent edition of Pozzi under the editorship of my friend, Dr. Brooks H. Wells, who has honored us with his presence at this meeting. They can be studied as well there as in this paper, the object of which is to discuss in a plain matter of fact way, but quite unsystematically, the importance of studying pelvic diseases early in their history, and in advising operation before irreparable mischief has been done.

Having now, in a very desultory way, called attention to the importance of early examination, early diagnosis and early operation in certain intrapelvic conditions,—having thus, as it were, furnished the motive for interference,—I may be pardoned for speaking somewhat more explicitly as to certain of the methods to be employed, and of the conditions to be defined. I have already said that I sometimes operate simply upon the history of the case and in the absence of physical indications, and I have given illustrative histories and illustrated results. It is unnecessary to discuss further that phase of the question. But what physical indications should prompt the attending physician to say to his patient that she has disease within the pelvis requiring surgical interference, and by what means will he arrive at his diagnosis? The last part of the question is the first in the logical order.

It is frequently most convenient to examine a patient at her own home, and an ordinary bed is then most generally employed for the purpose. It may be politic to acquiesce for the time being in this arrangement, especially in the cases of timid women who are being examined for the first time, and particularly as a presumptive preliminary diagnosis may be arrived at by this means. A final diagnosis, however, one embracing a careful study of the conditions within the pelvis, should not be attempted until after the patient has been placed upon a firm table in such position that the bimanual method can be satisfactorily practised. For an operator of experience, whose touch is cultured, it is not necessary to place the patient in any other than the recumbent posture with the knees flexed. The Sim's position may, in exceptional cases, be found to be of real value. The knee-chest and other postures sometimes affected have never been necessary in my practice. A speculum need not be employed. It is not infrequently resorted to *pro forma*. In the diagnosis of conditions above the pelvic diaphragm, it has no place. So manifest is this fact that many may wonder why it is given mention in this paper, but this same "many" may not know that there is another "many" by whom the speculum is employed just as if the uterine canal were a telescope, or the pelvic diaphragm were a plate-glass window. Instrumental examination of the uterine cavity is always hazardous, ought not to be employed in any but exceptional cases, and then only under the most scrupulous aseptic precautions.

Many a patient has died while her attending physician has frittered away time in a futile attempt at a differential diagnosis. There recently came into my consultation room a physician whose appearance and position would suggest that he had judgment and sagacity, but who at once dispelled the illusion by stating that he had a patient whom he should send in for operation just so soon as he could tell whether she had a pyosalpinx or a hydrosalpinx!

What difference would it make ? Suppose the probabilities of the case had been broadened, and had included follicular degeneration of the ovaries, chronic occlusive salpingitis, or any other or all of half a dozen other conditions, what difference would it have made? There can be but one line of treatment for them all.

It cannot be expected within the limits of a paper such as this that the physical indications of the various intrapelvic diseases can be outlined. Tumefaction above either fornix with or without fixation, generally means irreparable organic mischief, which, if not already furnishing a nidus for pus, offers, at least, an inviting field for suppuration. When this condition exists in connection with a history of previous miscarriages, dirty accouchments, intrauterine tinkerings, and electrical treatments, but particularly when a distinct history is given of one or more attacks of pelvic peritonitis, which, however, may not have been of a severe type, the conclusion is simply irresistible that the case belongs in the surgical category, and that operation should be advised and practised at the earliest possible moment.

311 Elm Street.

DISCUSSION.

Dr. H. D. Ingraham, of Buffalo : I am sorry that I was unable to hear Dr. Ross's paper upon ectopic gestation, as it is a very interesting subject, one that is of great importance to the general practitioner, at least as far as making the diagnosis is concerned. From the number of cases that I have seen, there can be no doubt that this condition occurs much more frequently than is generally believed by the profession. Although the other papers are much alike, they cover a very extensive field, one too broad to be taken up in the limited time allowed for discussion. I fully believe in operation when any distinct lesion exists that cannot be relieved by less radical measures. I don't believe it right to let women suffer, yet we are all well aware that many of the conditions that were formerly supposed to demand operation can be relieved without it.

I wish to add what emphasis I can to one point mentioned in one of the papers, and that is the necessity of early diagnosis in all diseases peculiar to women, especially that terrible one, uterine cancer. A great many cases of this disease are sent to the hospital with which I am connected, but most of them are so far advanced that nothing can be done for their relief. Delay has destroyed all hope, but had they been seen earlier much suffering could have been avoided and many years added to theirs lives. The same can be said of several other diseases.

I also wish to protest against the frequent and unnecessary use of the uterine sound. I do not go quite as far as Dr. Reed, because I have a sound and use it occasionally. Yet it is an instrument that does much more harm than good, unless very carefully and sparingly used. Several times during the past Winter I have seen men, who ought to know better, introduce the sound into a retroflexed and adherent uterus, and attempt to replace it, the only effect being to increase the suffering of the patient and prolong her illness. In my lectures I tell the students never to use the sound until they have been in practice three years, and then, if they use it, to be very cautious. If they go without it for that length of time, they will find that it is not very necessary, and will not, I believe, be likely to abuse it. I wish to join with the other members of the society in thanking our visitors for coming here and giving us such a treat.

Dr. Brooks H. Wells, of New York : Judging from the remarks of the previous speakers, one would suppose that nearly every case of salpingitis demanded a laparatomy. Dr. Reed says, however, that he only performs abdominal section on about twenty-five per cent. of his cases. I would like to ask what becomes of the other seventy-five per cent.? That certainly is an important majority that ought not to be entirely overlooked.

No one doubts the necessity for operation in the cases recorded by the previous speakers, who are all expert diagnosticians and surgeons, but others with less experience might be led to think themselves justified in removing appendages in any case where they found tubal enlargement and pain.

In my experience, most cases of catarrhal salpingitis, or hydrosalpinx, can be practically relieved by treatment short of section. Undoubted pus tubes, with peritoneal leakage and recurrent inflammation, should, with our present knowledge, be promptly removed. Yet, even after an undoubted gonorrheal double pyosalpinx, it is possible that a patient can be cured and bear children. My position in this matter is best illustrated by reference to a somewhat exceptional case where I treated the wife of a drummer,—with an acute gonorrhea contracted from her husband, and an acute periuterine inflammation, with great enlargement of the tubes,—first for the acute symptoms, and later, by careful dilatation, scraping and packing of the uterine cavity for the relief of very profuse menstruation. The packing caused no unpleasant symptoms, but was unexpectedly followed by a flow of pus from the uterine cavity, which continued at intervals for some time, and was accompanied by the disappearance of the tubal swelling, to be followed, six months later, by pregnancy. The patient was delivered in January last of a healthy girl, and had a normal convalescence.

Dr. Henry R. Hopkins, of Buffalo : I desire, before all else, to express my earnest appreciation of the papers and discussion. Permit me, as a general practitioner, to say that I consider the subject most practical, and its treatment in the highest degree edifying.

I regret my inability to add positively to the interest of the occasion, but I thought possibly some good might come from the observations of a general practitioner during, or resulting from, a quarter of a century's experience in cases of intrapelvic inflammatory troubles. But a single observation would I make, and that to the effect that, excepting cases where intrapelvic inflammations result from puerperal or gonorrheal infection, or from the equally mischievous and much more frequent meddlesome amateur gynecologist, Nature, in the majority of cases, is competent to bring the inflammatory process to a favorable termination without serious impairment in a permanent degree of either local function or health, provided the general health of the individual can be maintained at a fair standard. This observation, it seems to me, is fully supported by my experience.

Dr. H. E. Hayd, of Buffalo: Papers like Dr. Ross's only men of Dr. Price's, Dr. McMurtry's, and Dr. Reed's experience can thoroughly discuss, but these last three papers come within the province of us all. First of all, I want to thank our distinguished guests for the interesting papers they have given us this afternoon, and to assure them that they couldn't have chosen subjects which would have interested, instructed, and entertained so many of us as these papers have. Dr. Price, in his usual and characteristic manner, reviews the recently accepted ideas of uterine pathology, backed up by his personal observations and unusually brilliant experience. He has shown us that the old pelvic cellulitis, the broad ligament phlegmon, the para- and perimetritis are but manifestations of the same inflammatory process which has extended by contiguity from the uterine mucous membrane to the tube and its adjacent structures. He has pointed out a very important clinical fact, namely, to relieve endometrial troubles as quickly as may be, and by means as unirritating as possible.

His paper has been well complemented by Dr. McMurtry's, who has shown us the causal relationship between minor gynecological operations and severe uterine disease. He has brought to our minds personal experiences—perhaps not recent—of severe inflammations following the careless and indiscriminate use of the uterine sound in elevating a retroflexed organ, or the unnecessary measuring of the length of the uterus. No two papers could have done this society more good, and I congratulate our visiting doctors for coming among us in this missionary service, because every day those of us who see much uterine disease are struck with the boldness and the ignorance of men who use sounds, probes, and other appliances in the uterine cavity without the remotest idea of their danger. Admitting, therefore, the possibilities of great danger in their manifestations, there is still another side to this question, and that is the assurance of great benefit from properly directed intrauterine treatment in suitably selected cases, as was brought out by Dr. Wells. We come to Dr. Price and to Dr. McMurtry, and to Emmet, if he were present with us,—since he, above all others, deprecates the practice of invading the sacred precincts of the uterus for a supposed endometritis — and we tell them that rest, sexual abstinence, the elevated bedstead, the knee-elbow position, hot water injections, and glycerine tampons do not cure - although they help—the profuse discharges and the recurrent hemorrhages, the backaches and stomach reflexes, which so many of our women complain of, due to subinvolution, endometritis, metritis, and their associated salpingites; and we bring to them positive proofs and personal experiences of cures with the judicious intrauterine use of tincture of iodine, tincture of iodine and carbolic acid, and galvanism; and in more severe cases, forcible dilatation, currettement and drainage by iodoform gauze, as practised and recommended by Polk, of New York. Of course, we don't presume that cases such as are shown by Dr. Price of big sausage-shaped tumors and ovarian abscesses are relieved by these means, as nothing short of an operation could be indicated in such conditions, but we do maintain that this advanced state of inflammation and disorganization might have been prevented by the timely and judicious employment of these minor gynecological procedures. We wish to join with them in keeping up this cry of unnecessary uterine tinkering, and over-treatment of women who are apt to ascribe all their diseases to a tipped womb, when we know there are other organs capable of producing distressing symptoms than merely the uterus and its appendages.

Dr. Joseph Price, of Philadelphia: The thought suggests itself that a good motto for our office walls and those of the assembly rooms of our conventions, would be the kindly, thoughtful utterance of the good Quaker lady: "I shall pass through this world but once. Any good thing, therefore, that I can do, or any kindness that I can show any human being, let me do it now. Let me not defer it or neglect it, for I shall not pass this way again." We will make use of the opportunities of today, for we may not pass this way again.

The classical productions of Burnutz and Goupil, followed by the masterly teaching of Mr. Tait, in the line of pathological and anatomical research, have served as a great stimulus to the student who is eager in the pursuit of knowledge. Their arguments have the merit of almost mathematical accuracy and directness, and are reinforced on all sides by post-mortem examinations, together with careful notes of clinical histories. Within the last decade, more than ever before, the ranks of the profession have contained many men who have entered the field of research, eager and anxious and willing to toil in mining out the best treasure of scientific truth. Much valuable teaching, from a clinical and scientific standpoint, has been and is being done. The practical lessons being learned at our operating tables will lead to the revising of much of our old literature, and the teaching of a better and more prompt surgery. The earnest, truth-seeking, hard-working men of the profession have learned, through the open avenues of surgical observation and experience, that there has been much erroneous and dangerous teaching in regard to a great variety of pelvic troubles. Now, with almost perfect opportunities of obtaining exact knowledge of the natural history of pelvic maladies, of the angry forms of tubal and ovarian disease, there exists no rational excuse for much of the ignorance existing and put in practice by men who would assume the office of teachers. We assume to lay down in no line of our work absolute directions. None of us "know it all," and it will be a long time before we do. Our advancing steps will be greatly facilitated by a close watchfulness for our own and others' errors, and by the tenacious holding on to that only which has the strong support of happy results.

In the light of our present clinical knowledge, acquired by large experience in the pelvis, we are driven to the conclusion that the entire subject of pelvic peritonitis and cellulitis will have to be rewritten. Sharp distinctions between pelvic cellulitis and peritonitis are refinements of diagnosis that we no longer seek. Our old literature is crowded full of grave discussions of pelvic troubles, under such captions as peritonitis, parametritis, pelvic abscess, and the like, with lengthy disquisitions as to pathological changes, very *lucid* scientific explanations of the causes of the conditions, and the varieties of the treatment indicated in each.

To *blot out old useless traditions, to efface old errors and bad teaching* from many of our books, would leave very little book and less author, for, with but a few heroic exceptions, the author holds on to his old teaching of errors, and passes out of a living progressive world. That eminent author and scientist, Prof. Simpson, of Edinburgh, forcibly implied his opinion of much of what has been written when asked by the college librarian how he should rearrange the college library, he replied: "Put all the books over ten years old down in the cellar." This is taking hold of an important matter at the right end. Such a course with our own college and private libraries would have a chilling if not maddening effect upon the very refined sensibilities of some of our modern American authors. They would take it as *personal*, and as exhibiting the incendiary spirit of ignorance. But even in this line we would not be conservative. "The man who knows it all" is the man who makes no advances, who never labors to find new truths and better ways, who does not zealously seek all through his own experience for his own errors and profit by the lessons they teach, who never studies for his own and general professional success the lessons of the errors and victories of the students and toilers of the profession. He is one who wears his January moss through the hot June days, and takes it to be profanity to his sacred person if you breathe on it. There is nothing men who would learn welcome so much as criticism. The great giant master-mind and hand in abdominal surgery, Tait, comes to the fore again and again to tell us of his errors and how and wherein he corrected them. The men who have written the best are the men who, from their larger experience, are revising or making new editions, with very little of their old books in them. And they are not wasting time and energy in apologies. The man of the period we would have talk to us, is the man who comes to us with his apron on and with clean hands. He need not be precisely a Lord Chesterfield in his manners. The spider may have reached the perfection of his art in the weaving of his first web, but it has not been given to us to know all or attain perfection with so much facility and ease.

Perimetritis has been defined for us as "an inflammation of the peritoneal covering of the uterus and its appendages," a comparatively rare condition, frequently fatal. Parametritis, as "an inflammation of the cellular tissue of the broad ligament," a common condition, often ending in abscess, and rarely fatal; while "pelvic abscess means pus in the pelvis." Such are the ideas and teachings of the old, surgically orthodox school.

We believe them to be essentially salpingites, inflammation of the tubes and ovaries being the primary cause of these troubles. That peritonitis is commonly the result of tubal and ovarian disease, is one of the plain lessons of the operating table,—a conclusion of the best surgical experience.

The condition known as parametritis may possibly occur, and does occur, but secondarily to advanced tubal disease.

Inflammation of the cellular tissue and formation of pus can occur anywhere in the body where there is cellular tissue, from the scalp to sole of the foot. But we never see it in the pelvis independent of tubal trouble. If it ever does so occur, it must be the result of traumatism, and cannot fail to involve surrounding structures,—suppurating ovarian, or dermoid cyst, or salpingitis, caused by gonorrhea, parturition, injury to small tumors, dirty instruments, electricity, etc. We have seen pus discharging from the rectum, from the bladder, from the umbilicus, and from the vagina. We see psoas abscess, perforating appendicitis, idiopathic peritonitis, and typhoid fever, and find the origin of the trouble in the suppurating tubes and ovaries. Pus in the pelvis is very rarely a simple single abscess, and this fact has most important bearing in the treatment. From the anatomical relations of the organs, the complexity is easily understood. Peritoneal inflammations and adhesions are always present. The tubes are commonly strictured and full of multiple pus pockets, separate from each other and from other collections of pus, as pus pockets in the ovary or in the cellular tissue. The condition very frequently occurs on both sides of the uterus at the same time. There may be double pyosalpinx and double ovarian abscess contained in a pus pocket in the peritoneal cavity composed of adherent intestinal inflammatory tissue, four abscess cavities contained within a fifth. But I must give way to others who will continue the discussion.

Dr. Chas. A. L. Reed, of Cincinnati : It is difficult to discuss two such papers as those read respectively by Drs. Price and McMurtry, for they have left but little to be said upon the topics of which they treat. There is, however, one point in Dr. Price's paper which I wish not to discuss, but to emphasize, and that is that purulent accumulations within the pelvis and outside of the uterine appendages do occur, but as far as observation has gone, only as post-puerperal complications. This is precisely the position that I assumed before the American Association of Obstetricians and Gynecologists at New York a year ago. I then alluded to several cases in which I made median exploratory incisions, and had found the appendages, upon close and careful inspection, entirely free from disease, but large purulent deposits in the deep structures upon one, not both sides of the uterus, which deposits I evacuated by incision along Poupart's ligament. I have had three cases since that time. In every instance the accumulation was consequent upon and, logically, caused by puerperal infection. I recall that in several instances there existed yet uncicatrized lacerations of the cervix. This sort of traumatism opens the lymph channels to an infection which results in a circumscribed accumulation of pus in the deep tissues of the pelvis, but, so far as my limited experience goes, always upon one side of the uterus. Of course, it is but logical to infer that if this condition can and does result from a traumatism incident to labor, it may result from any other deep injury to the uterine tissues. My only reply to this is that any conception of pathology, or of pathological states arrived at deductively rather than by observation, is unreliable, and that my experience and my research, the latter giving discriminating weight to the respective authorities, confines this condition to just where Dr. Price has placed it, viz., in the category of post-puerperal complications.

I have been interested in the remarks of Dr. Crego, who has given the views of the neurologists upon the question of genital reflexes. I believe he has very accurately stated the case from that standpoint. And I feel the more interest in what he has said because, about three years ago, in this very hall, I had occasion to discuss that question at some length. I then assumed the position that mental and nervous diseases do sometimes occur as the result of intrapelvic disease in women, and that the removal of the intrapelvic disease resulted in the cure of the mental and nervous complications. I made that observation based upon some experience, which has since grown more extensive, and which has only confirmed the convictions I expressed at the time referred to. My quarrel, friendly of course, with the alienists and the asylum men is that they deny the lesson taught by this observation, and that they do think just as Dr. Crego has represented. Happily, however, the proof of the correctness of my position is coming, and coming rapidly. A gynecologist has at last been appointed the medical officer of a hospital for the insane. He has dispelled several illusions which have been imposed upon the profession and the community by the old and, unfortunately, yet existing *régimé*. In the first place, he has found that these patients can be examined without injury to themselves; in the next, that disease of the intrapelvic organs can be as readily diagnosticated in their cases as in others ; that they can be operated upon as readily and with as good results as sane patients ; and, finally, that after primary recovery from abdominal section, a very encouraging proportion of them recover mentally ; and, still lastly, that among the most happy mental recoveries are cases that have been incarcerated for years and labeled as incurable. I invite the attention of this society and of the profession to the magnificent work being done in this direction by my distinguished friend, Dr. George H. Rohé, of the Maryland Hospital for the Insane. When asylums become organized as hospitals, with staffs of specialists in the various departments in attendance, we shall realize the maximum of recoveries in these institutions.

[NOTE BY THE EDITOR.—Every exertion was made to obtain the remarks of Dr. Crego, referred to in Dr. Reed's discussion, but without avail.]

DR. MCMURTRY : Closing the discussion. It is not my intention by my paper to convey an idea that the minor gynecological operations are to be abandoned. On the contrary, I would advocate elevating the standard and improving the methods, and thereby the results of these valuable surgical procedures. But I would direct attention to the fact that when misapplied, or performed without observing a thoroughly surgical technique, these procedures, even though generally considered simple, innocent, and conservative, are capable of inflicting serious mischief upon the patient by establishing intrapelvic inflammation, or aggravating such a process already in existence. My plea is for more careful discrimination in diagnosis, more accurate ideas as to pathology, and the observance of established surgical methods when these operations are performed in appropriate cases.

The fact is established by careful clinical observations, confirmed at the operating table, that extreme degrees of tubo-ovarian disease may result from the misapplication of these apparently simple methods of treatment. Having learned this, it should be the aim of every practitioner to exercise greater care in diagnosis and more thorough discrimination in resorting to minor operations and local treatment; and, at the same time, to cultivate the same rigid observance of operative technique as in major operations involving the peritoneum. In the treatment of minor ailments, and in doubtful conditions, we should withhold our hand rather than expose the patient to greater danger by resorting to operations which have a strictly limited field of application.

